# Serial measurement of circulating cardiovascular-enriched microRNAs in patients with ischaemic heart disease – a five-year longitudinal study

**DOI:** 10.1042/BSR20253835

**Published:** 2025-12-23

**Authors:** Jayanthi Bellae Papannarao, Sean Coffey, Andrew Gray, Michael Williams, Rajesh Katare

**Affiliations:** 1 Department of Physiology,Faculty of Biomedical Sciences, University of Otago, Dunedin, 9010, New Zealand; 2 HeartOtago, Cardiovascular Research Centre, University of Otago, Dunedin, 9010, New Zealand; 3 Department of Medicine, Faculty of Medicine, University of Otago, Dunedin, 9010, New Zealand; 4 Department of Preventive & Social Medicine, Dunedin School of Medicine, University of Otago, Dunedin, 9010, New Zealand

**Keywords:** cardiac function, ischemic heart disease, microRNAs, prognostic marker

## Abstract

Patients with ischaemic heart disease (IHD) require frequent monitoring, as the transition from stable disease to acute life-threatening events remains largely unpredictable. MicroRNAs (miRNAs), small non-coding RNAs involved in physiological and pathological processes, are released into circulation and remain stable. In this study, we aimed to determine if the serial measurement of cardiovascular-enriched circulating miRNAs could reflect changes in cardiac function in patients with IHD. Fifty-three new IHD patients participated in a five-year study with regular echocardiography, blood tests and follow-ups at 12-month intervals, with 25 followed up at five years. Serial echocardiography revealed a pattern towards worsening diastolic function over the follow-up period. RT-PCR analysis was conducted on four cardiovascular-enriched circulating miRNAs: miR-1, miR-126, miR-132 and miR-34a. Among these, only miR-1 and miR-126 showed statistically significant down-regulation starting from 24 months onwards. Linear mixed models, adjusted for body mass index and HbA1c changes, indicated significant associations between fold changes in miR-1 and miR-34a with cardiac function changes. Results from this first-ever five-year follow-up study have identified a possible link between cardiovascular-enriched miRNAs, miR-1 and -34a and cardiac function in patients with IHD, providing a foundation for prognostic tests for IHD and laying the foundation for further studies in larger populations. It is noteworthy that a significant dropout of participants may have impacted the statistical power in our study.

## Introduction

The transition from a stable disease state to a life-threatening event in patients with ischaemic heart disease (IHD) remains unpredictable and can progress rapidly [1], especially in patients with underlying conditions like diabetes or hypertension [2]. Much of the morbidity and mortality seen in patients with IHD is related to the underlying cardiac function and, therefore, the identification of a sensitive and specific biomarker that can easily monitor cardiac function without relying on more resource-intensive assessment such as echocardiography would be highly advantageous.

MicroRNAs (miRNAs), small RNA molecules involved in gene expression regulation, have shown promise as potential prognostic markers [3]. miRNAs have been increasingly recognised as critical regulators of cardiovascular health [3]. In support of this observation, we recently showed a direct correlation between the expression of three cardiovascular-enriched miRNAs, which play a significant role in cardiac homeostasis and the development of cardiovascular dysfunction in the hearts of type-2 diabetic mice [4–6]. Specifically, we and others have demonstrated dysregulation of miR-1 that is associated with cardiac conduction [5,7], miR-126 associated with vascular homeostasis [4,8], miR-132 associated with hypertrophy [9] and miR-34a [6,10], that is associated with accelerating senescence and apoptotic cell death in diabetes and IHD. Furthermore, clinical studies have revealed a strong association between circulating cardiovascular-specific miRNAs and ejection fraction (EF), a clinical measure of heart contractility [11]. To date, there is little information regarding the role of circulating miRNAs as markers of cardiac function in chronic IHD.

## Hypothesis

We hypothesise that circulating cardiovascular-enriched miRNAs exhibit dynamic changes corresponding to the progression of IHD and that these alterations are significantly associated with changes in cardiac function as assessed by echocardiography. Furthermore, serial measurement of these circulating miRNAs may serve as a valuable non-invasive prognostic biomarker for monitoring disease progression and functional decline in patients with IHD.

## Methods

### Ethics and participant recruitment

The study complies with the *Declaration of Helsinki*, and the research protocol was approved by the Human Ethics Committee of the University of Otago, New Zealand. All the participants signed an informed consent form and were given the opportunity to withdraw from the study anytime. To investigate the potential of miRNAs as prognostic markers in IHD, we conducted a five-year longitudinal study involving newly diagnosed IHD patients (*n*=53 recruited, 16 discontinued after 12 months, 2 died, contact was lost for 2, 8 completed partial follow-up and 25 completed full 60 month follow-up). The mean ± SD age of the participants at baseline was 61.7 ± 9 years (44 males and 9 females, Table 1). Out of the total participants, 22 were diabetic (15 male and 7 female). The study design is summarised in Supplementary Figure S1.

**Table 1:** Table showing participants' characteristics.

Participants' characteristics irrespective of diabetes							
No. of participants recruited: 53 (22 diabetic) male: 44 (15 diabetic) female: 9 (7 diabetic)							
Characteristics	Baseline (*N*=53)	6 months (*N*=53)	12 months (*N*=53)	24 months (*N*=34)	36 months (*N*=28)	48 months (*N*=28)	60 months (*N*=25)
Age (years)	61.7 ± 9	62.1 ± 9.2	62.6 ± 9.1	63.9 ± 8.6	65.2 ± 8.4	66.4 ± 8.4	67.5 ± 8.9
E/e’	9.7 ± 3.6^a^	9.4 ± 3.3^b^	10.0± 3.5^c^	9.8 ± 3.4^d^	10.2 ± 2.7^e^	10.3 ± 3.1 ^e, n^	10.7 ± 4.3^f, o^
E vel (m/s)	0.68 ± 0.15^k^	0.67 ± 0.15^c^	0.68± 0.14^l^	0.67 ± 0.15	0.67 ± 0.16^e^	0.65 ± 0.17 ^e, p^	0.67 ± 0.20^m^
IVSd (cm)	0.97 ± 0.19	0.97 ± 0.21	0.97 ± 0.22	1.01 ± 0.23	1.01 ± 0.24	1.03 ± 0.21^q^	1.03 ± 0.24^f ,r^
FS (%)	32.1 ± 6.9^g^	31.1 ± 5.6^c^	31.1 ± 5.6	30.7 ± 5.5	29.2 ± 5.6^h^	32.5 ± 5.9	31.7 ± 5.6^i^
EF (%)	56.1 ± 5.9^g^	56.0 ± 7.7	55.6 ± 7.0^c^	54.2 ± 7.2	54.5 ± 5.1^j^	55.4 ± 7.6	56.0 ± 7.6^f^

Note that some echocardiography data were not available for all the participants due to the way the echocardiography was analysed by the sonographer (^a^ *n*=46, ^b^ *n*=45, ^c^ *n*=52, ^d^ *n*=33, ^e^ *n*=28, ^f^ *n*=23, ^g^ *n*=50, ^h^ *n*=27, ^i^ *n*=21, ^j^ *n*=26, ^k^ *n*=51, ^l^ *n*=53, ^m^ *n*=25). ^n^ *P*=0.0691, ^o^ *P*=0.2943, ^p^ *P*=0.078, ^q^ *P*=0.024 and ^r^ *P*=0.189 versus baseline using exact Wilcoxon signed-rank tests. All the data are mean ± SD.

BMI, body mass index. EF, ejection fraction. E vel, E velocity. FS, fractional shortening. IVSd, interventricular septal thickness during diastole.

### Sample collection and echocardiography

Following informed consent, blood samples were collected from the participants at the time of initial diagnosis and at 6, 12, 24, 36, 48 and 60 months after treatment initiation to measure four cardiovascular-enriched miRNAs (miR-1, -126, 132 and -34a, target miRNAs). Echocardiography was performed at each time point to assess changes in cardiac function.

### Total RNA extraction and RT-PCR analysis

Target miRNA expression was determined using real-time PCR analysis as described in our previous studies [4,12]. In brief, total RNA was extracted from plasma using the Qiagen miRNeasy Mini Kit (Qiagen). The concentration of the extracted RNA was measured using a NanoDrop 3000 spectrophotometer (ThermoFisher). To determine the expression levels of the target miRNAs, 20 ng of total RNA was reverse transcribed using specific stem-loop reverse transcription primers for each target miRNA, followed by amplification using specific TaqMan hybridisation probes. miR-16 and miR-24 were chosen as endogenous control miRNAs [13]. The relative delta cycle threshold was calculated by normalising the CT of the target miRNAs to the average CT of the endogenous controls.

### Statistical analysis

Statistical analysis for RT-PCR data were conducted using GraphPad Prism software. Data were analysed using one-way ANOVA, mixed-effect analysis with Giesser–Greenhouse correction and uncorrected Fisher’s LSD with individual variances computed for each comparison. The difference between non-diabetic and diabetic groups was analysed using one-way ANOVA, mixed-effect analysis with Giesser–Greenhouse correction and uncorrected Fisher’s LSD with individual variances computed for each comparison. Associations between each miRNA and each cardiac function measure were examined using linear mixed models with a random participant effect to accommodate the multiple measurement periods and with restricted maximum likelihood (REML) used to estimate effects. This was done using concurrent changes in the miRNA for each period (baseline to 6 months, 6 to 12 months, 12 to 24 months, 24 to 36 months, 36 to 48 months and 48 to 60 months) and using changes in miRNA from the preceding period. Models were fitted without and with adjustment for changes in body mass index (BMI) and HbA1c during the same period.

## Results

**Figure 1:**
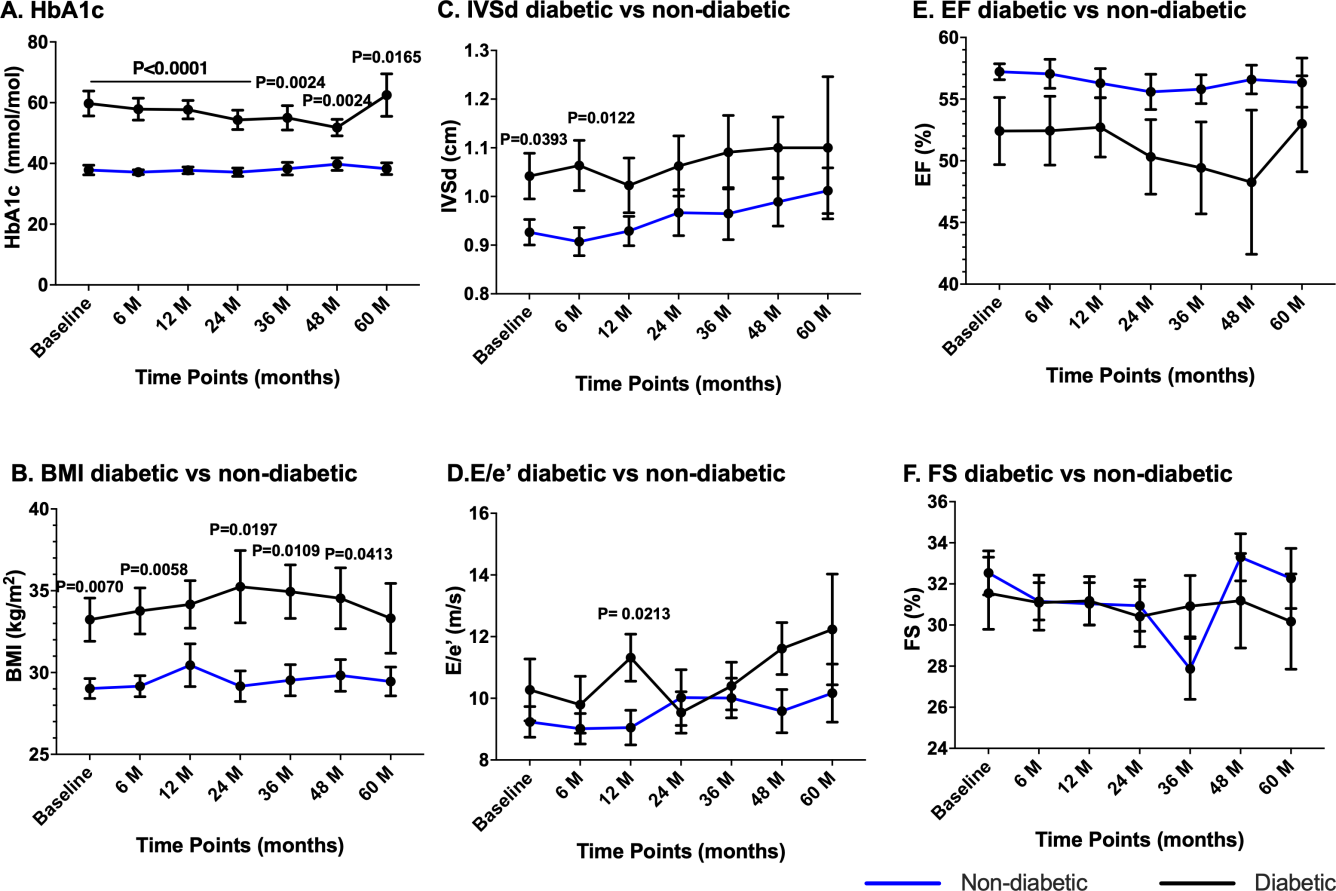
Cardiac function, HbA1c and BMI over the course of the study. Line graphs showing the changes in glycated haemoglobin (HbA1c, **A**), body mass index (BMI, **B**), intraventricular septal thickness at diastole (IVSd, **C**), E/e’ (an indicator of diastolic function, **D**), ejection fraction (EF, **E**), and fractional shortening (FS, **F**) at different time points in diabetic and non-diabetic participants. Data were analysed using one-way ANOVA, mixed-effect analysis with Giesser–Greenhouse correction and uncorrected Fisher’s LSD with individual variances computed for each comparison. Statistical analysis presented in the figure is versus the corresponding time point in non-diabetic participants. It is to be noted that participants were at different ages at each timepoint and are of different sex distributions; this was not accounted for this analysis.

All the general information and individual echo data of participants are in Supplementary Table S1. Out of the total participants, 22 were diabetic (15 male and 7 female). Diabetic participants had significantly higher HbA1c (Figure 1A) and BMI (Figure 1B) compared with non-diabetic participants. However, there was no time-dependent effect on HbA1c and BMI in both diabetic and non-diabetic participants (Figure 1A&B). Echocardiography measurement showed a significant difference in interventricular septal thickness during diastole (IVSd) (Figure 1C) and E/e’ (Figure 1D), the indicators of wall thickness and diastolic function, respectively, between diabetic and non-diabetic in the initial stages of the IHD; however, this disappeared with time (Figure 1C&D). There were no changes observed in ejection fraction (EF), a measure of contractility, both between diabetic and non-diabetic groups over time (Figure 1E). When the data were analysed together irrespective of diabetes, both IVSd and E/e’ showed a pattern towards increase at 48 and 60 months (Table 1).

**Figure 2:**
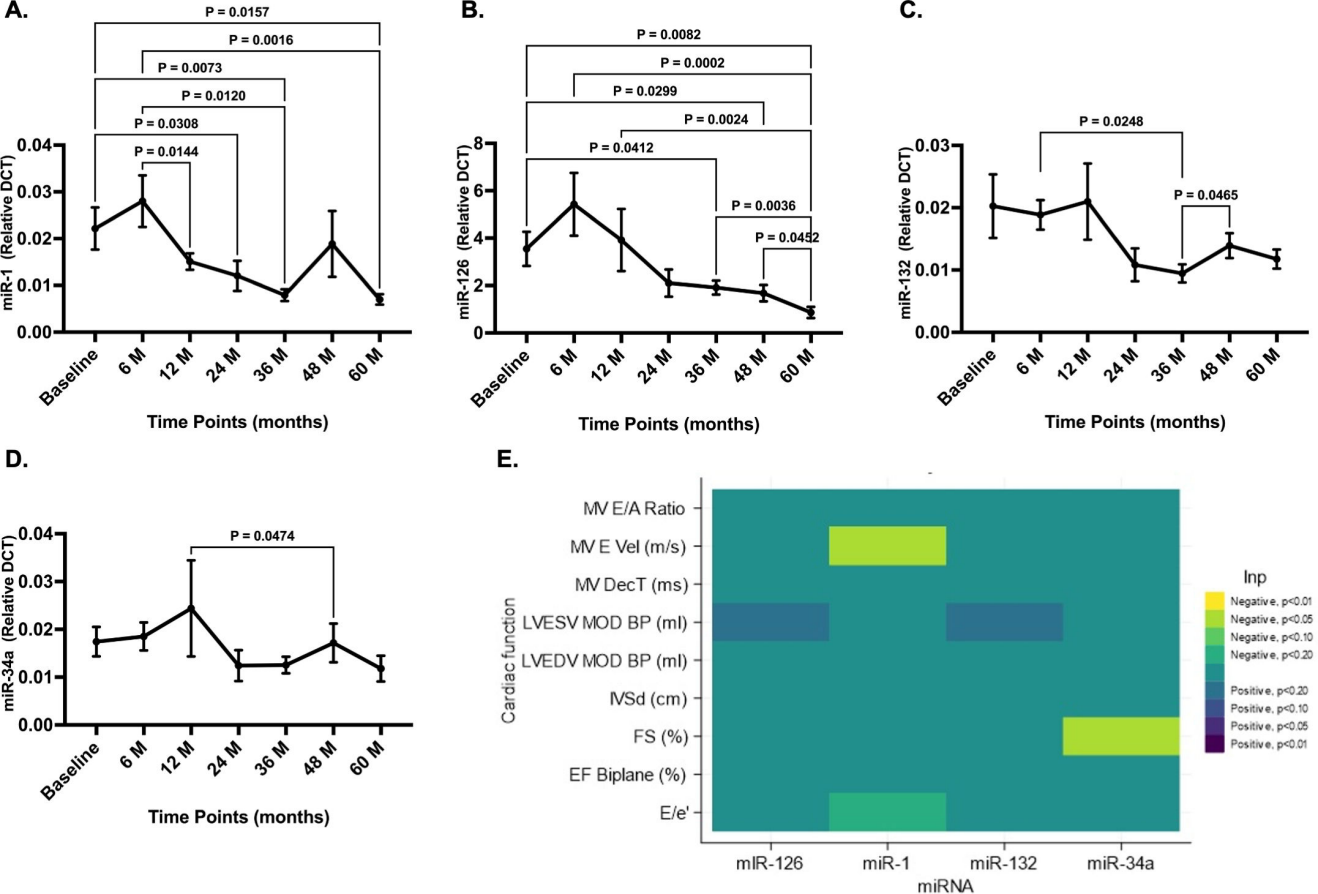
Changes in the miRNA expression. A–D. Line graphs showing the expression of target miRNAs at different time points. Pro-survival miR-1 and pro-angiogenic miR-126 showed an initial up-regulation at 6 months follow-up. This is likely due to the compensatory mechanism of the heart in response to ischaemia. With the progression of the disease, miR-1, -126 and pro-hypertrophic miR-132 were down-regulated. While we expected to observe up-regulation of pro-senescent miR-34a, there was no significant change in this miRNA expression. Data were analysed using one-way ANOVA, mixed-effect analysis with Giesser–Greenhouse correction and uncorrected Fisher’s LSD with individual variances computed for each comparison. **E.** Heatmap showing *P*-value and direction for associations in adjusted, concurrent model. Results showed a significant negative associations between miR-1 and MV E velocity (m/s) (*P*=0.026) and miR-34a and fractional shortening (FS%), *P*=0.048.

RT-PCR analysis of target miRNAs irrespective of diabetes showed an initial up-regulation of pro-survival miR-1 (Figure 2A) and proangiogenic miR-126 (Figure 2B) at the 6-month follow-up. However, with disease progression, the expressions of miR-1, miR-126 and pro-hypertrophic miR-132 were down-regulated (Figure 2A–C). There was no change in the expression of pro-senescent miR-34a over time (Figure 2D). Interestingly, there was no difference between diabetic and non-diabetic (Supplementary Figure S2) or hypertensive and non-hypertensive participants except at the 24-month timepoint for diabetic participants (Supplementary Figure S3).

Associations between each miRNA and each cardiac function measure were examined using linear mixed models with a random participant effect to accommodate the multiple measurement periods and with REML used to estimate effects. This was done using concurrent changes in the miRNA for each period (baseline to 6 months, 6 to 12 months, 12 to 24 months, 24 to 36 months, 36 to 48 months, and 48 to 60 months) and using changes in miRNA from the preceding period. Models were fitted without and with adjustment for changes in BMI and HbA1c during the same period.

None of the lagged models found statistically significant associations (all *P*≥0.126). From the concurrent models, two miRNA were found to be associated with cardiac function: miR-1 and mitral valve (MV) E Velocity in the unadjusted (*P*=0.045) and adjusted models (slope=−0.004, 95% CI −0.007 to −0.000, *P*=0.026) models and miR-34a and fractional shortening (FS%) in the adjusted model (slope=−0.151, 95% CI −0.301 to −0.001, *P*=0.048) but not the unadjusted model (*P*=0.073) (Figure 2E).

## Discussion

This five-year longitudinal study has established a link between circulating miRNAs and cardiac function in patients with IHD. Our findings reveal significant correlations between miR-1 and MV E velocity, as well as miR-34a and FS%, suggesting that these miRNAs can serve as potential biomarkers for monitoring cardiac function. In support of this, previous studies have demonstrated a strong association between miR-1 and diastolic function [7] and miR-34a and systolic function [14]. Although not statistically significant, the observed trend towards up-regulation of pro-survival miR-1 and proangiogenic miR-126 at 6 months could be speculated as a possible heart’s compensatory response to ischaemia; however, this needs further investigation. However, the subsequent down-regulation of miR-1, miR-126 and pro-hypertrophic miR-132 with disease progression highlights the dynamic nature of miRNA expression in response to chronic cardiac stress. A small sample number is a limitation for this study due to unexpected withdrawal and death of participants during the study. Another possible limitation could be the medications prescribed to the participants, which could potentially influence miRNA expression. However, with the limited number of samples, it was not possible to match the participants with similar medications. Nevertheless, our study supports the potential of miRNAs as a non-invasive assessment of cardiac function following a diagnosis of IHD, offering a potentially valuable tool for early detection and monitoring of potentially modifiable changes. Future research with larger cohorts is essential to validate these findings and to explore the mechanistic pathways through which these miRNAs influence or reflect cardiac function.

## Future directions

Building on the novel findings from this five-year longitudinal study, future research should focus on validating the prognostic value of circulating miR-1 and miR-34a in larger and more diverse populations with IHD. These studies should evaluate the consistency of associations in different clinical settings and analyse results based on factors such as age, sex, comorbidities like diabetes and hypertension, and therapeutic interventions. Further, expanding the range of analysed miRNAs and integrating longitudinal miRNA profiling with transcriptomic and proteomic data could further improve diagnostic accuracy and prognostic significance. Furthermore, to facilitate clinical application, developing standardised protocols for miRNA quantification, normalisation and interpretation will be essential for incorporating these biomarkers into routine cardiovascular monitoring and risk stratification strategies.

## Supplementary material

online supplementary figure 1.

online supplementary table 1.

## Data Availability

All data generated or analysed during this study are included in its supplementary information files.

## Abbreviations

BMI: body mass index
EF: ejection fraction
FS: fractional shortening
IHD: ischaemic heart disease
IVSd: interventricular septal thickness during diastole
MV: mitral valve
REML: restricted maximum likelihood
miRNAs: microRNAs
