# Supplementary material for: Serial measurement of circulating cardiovascular-enriched microRNAs in patients with ischaemic heart disease – a five-year longitudinal study
Source: Biosci Rep. 2025 Dec 23;45(12):809–14. doi: 10.1042/BSR20253835 (PMC12865885; doi:10.1042/BSR20253835)
Supplement: online supplementary figure 1. [file bsr-45-12-BSR20253835-s001.pdf]

Supplemental Figure 1

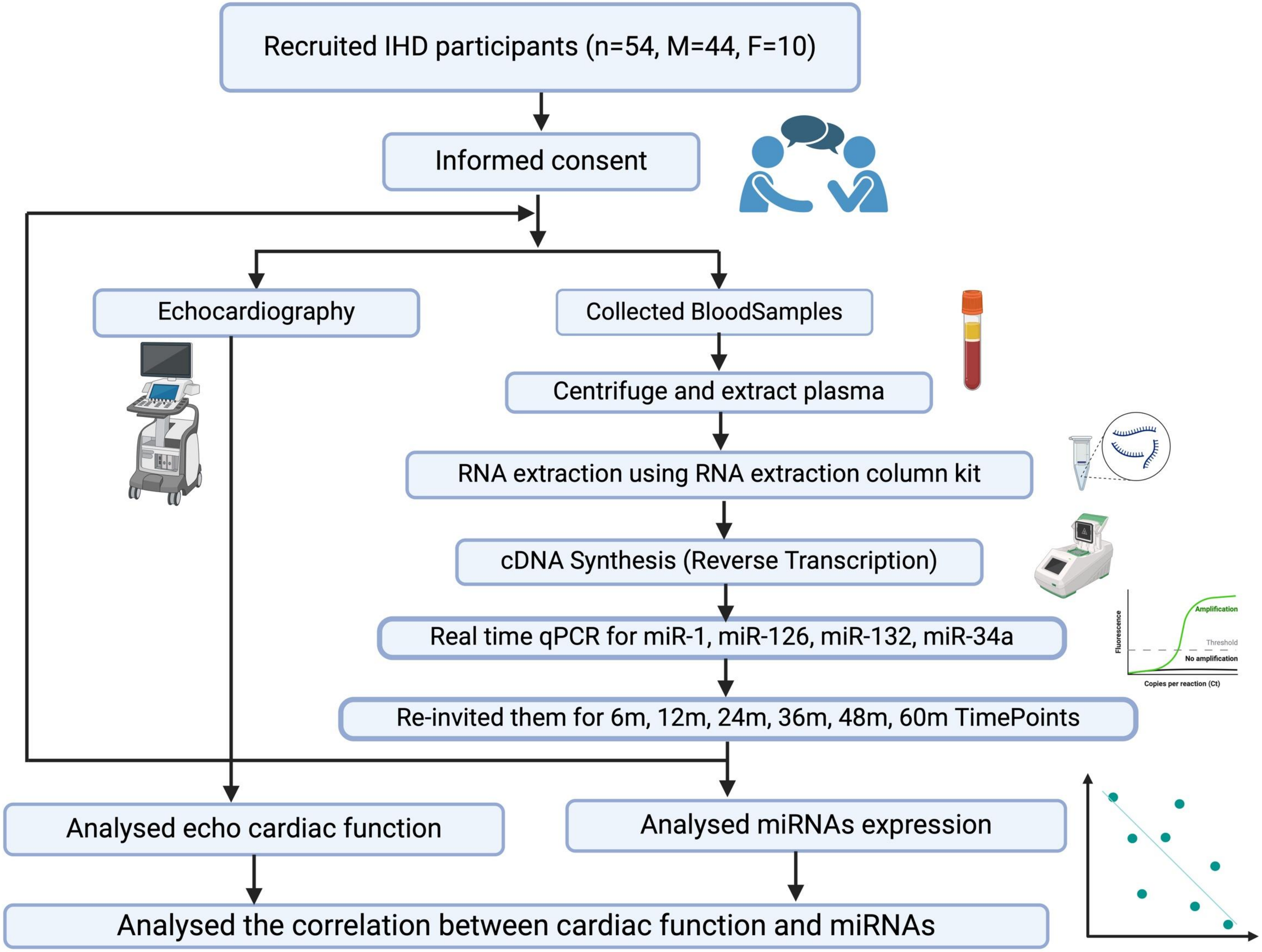

Supplemental Figure 1: Summary of the study design. IHD – ischemic heart disease; qPCR – quantitative PCR; miRNA – microRNA.

Supplemental Figure 2

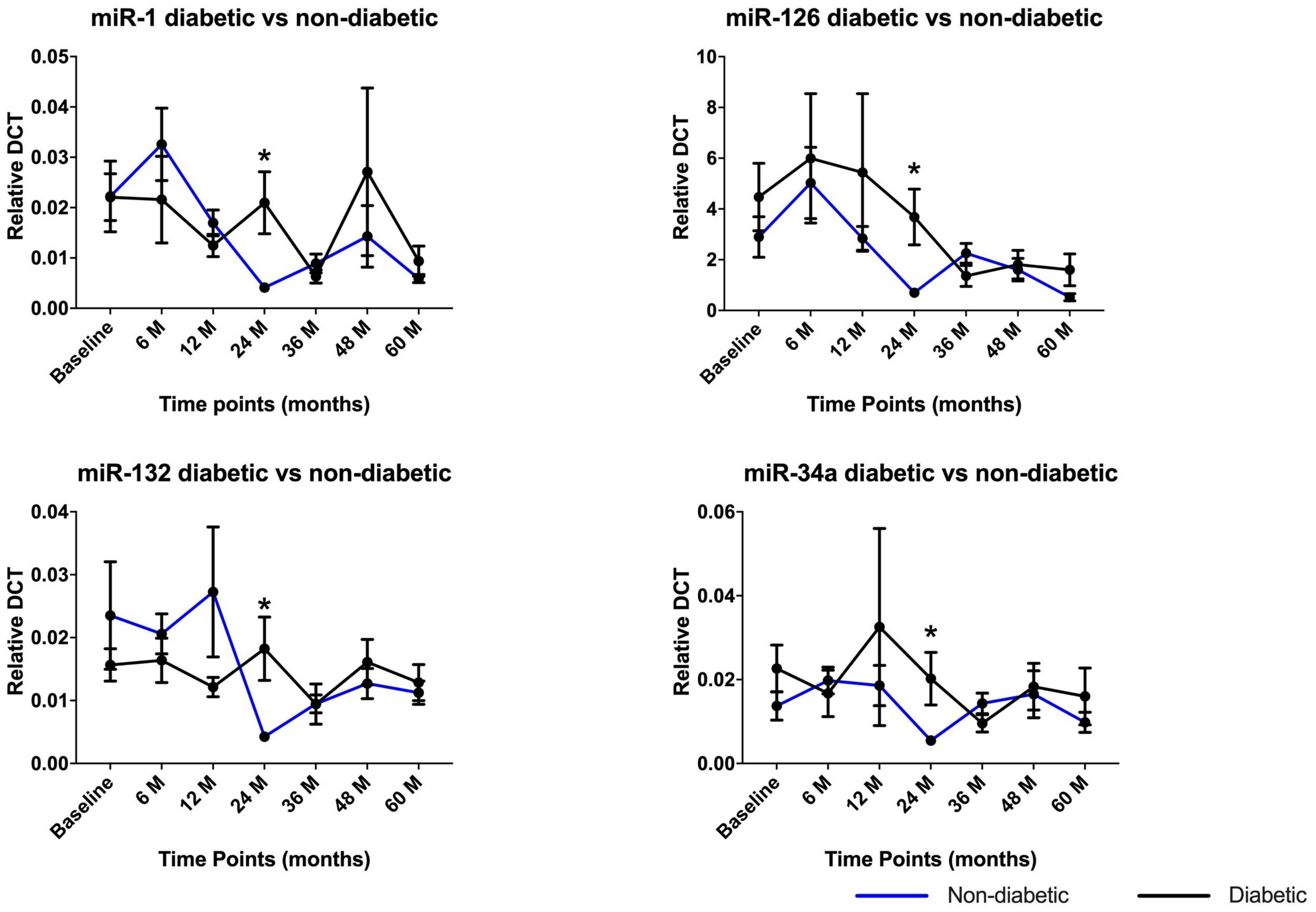

Supplemental Figure 2: Line graphs showing the expression of target miRNAs at different time points between diabetic and non-diabetic participants. Data were analysed using one-way ANOVA, mixed-effect analysis with Giesser-Greenhouse correction, uncorrected Fisher's LSD, with individual variances computed for each comparison. *P < 0.05 vs corresponding time point in non-diabetic participants. It is to be noted that participants were at different ages at each timepoint and are of different sex distributions, this was not accounted for this analysis.
